# Intervention of Compound Xueshuantong Capsule on the incidence of heart failure in patients with acute myocardial infarction after PCI based on the combination of disease and syndrome: A multi-center, randomized, double-blind, controlled trial

**DOI:** 10.1097/MD.0000000000032311

**Published:** 2022-12-16

**Authors:** Xiao-Dan Yang, Jia-Xi Shi, Wei-Can Liao, Jia-Yan Cui, Zheng Jin, Dong-Liang Liu, Xin-Lin Chen, Rong Li, Hui Wu, ChuanJin Luo, QingMin Chu, Rui Li, Wei Wu, LiJin Qing

**Affiliations:** a Clinical Medical College, Guangzhou University of Chinese Medicine, Guangzhou, Guangdong, China; b Zhujiang Hospital of Southern Medical University, Guangzhou, Guangdong, China; c Guangdong Zhongsheng Pharmaceutical Co., Ltd, Dongguan, Guangdong, China; d Basic Medical Science College, Guangzhou University of Chinese Medicine, Guangzhou, Guangdong, China; e The Affiliated Hospital of Guangzhou University of Chinese Medicine, Guangzhou, Guangdong, China.

**Keywords:** Compound Xueshuantong Capsule (CXSTC), post-infarction heart failure, randomized controlled trial

## Abstract

Heart failure (HF), manifested as a severe or end stage of various cardiac diseases, is characterized by increased incidence, mortality, re-hospitalization, and economic burden. Myocardial infarction (MI) is one of the most common and important causes of HF. Since 2005, acute MI (AMI)-associated mortality in China has been on the rise, and MI accounts for 23.1% of the causes of HF. Traditional Chinese medicine (TCM) has the unique advantages of controlling angina pectoris and HF symptoms, and improving patients’ quality of life. Compound Xueshuantong Capsule (CXSTC), also named as Fufang Xueshuantong Capsule, has the effect of increasing cardiac output and protecting myocardial function. In this trial, we aim to investigate the efficacy and safety of CXSTC in the prophylactic treatment of post-infarction HF and attempt to provide a clinical evidence-based basis for the prophylactic treatment of HF after AMI using TCM.

**Methods:**

This will be a multi-center, randomized, double-blind, placebo-parallel controlled trial. A total of 300 patients diagnosed with AMI and undergoing percutaneous coronary intervention within 12 hours of diagnosis will be randomized 1:1 into 2 groups: the control group that will be administered conventional Western medicine plus placebo and the trial group that will be administered XST along with the conventional Western medicine. The duration of treatment will be 3 months and the follow-up will be up to 6 months for both groups. The main efficacy indicator is the incidence of HF. The secondary efficacy indicators are cardiac function classification, 6-minute walk test score, TCM syndrome score, survival quality score, brain natriuretic peptide level, ultrasensitive C-reactive protein level, and cardiac ultrasound result. Data will be collected to analyze the underlying mechanisms by using IBM SPSS 23.0 software.

**Discussion:**

By investigating the efficacy and safety of CXSTC, this study will provide a clinical evidence base for the use of TCM in the prophylactic treatment of post-infarction HF.

## 1. Introduction

Post-infarction heart failure (HF) is when HF occurs after an acute heart attack (including ST-segment elevation myocardial infarct and non-ST-segment elevation myocardial infarct).^[1]^ Previously, a few China-HF studies showed that 11.2% of patients with HF had an obsolete heart attack.^[2,3]^ The Japanese Acute Heart Attack Registry Study found that among patients with ST-segment elevation myocardial infarction (MI) undergoing percutaneous coronary intervention (PCI), the readmission rate was higher among patients who developed HF than in those who did not experience HF within the first year.^[4]^ Numerous studies have shown a significantly higher risk of HF in patients after an acute heart attack than in patients who did not have a heart attack.^[5–8]^ The key to treating post-infarction HF mainly involves blocking the overactivation of the neuroendocrine system. Studies have shown that β-blockers and angiotensin-converting enzyme inhibitors (ACEIs) can improve survival in patients with MI but they require long-term treatment with the maximum tolerated doses.^[9]^ Owing to adverse effects such as electrolyte disturbances, renal insufficiency, and hypotension, patients find it challenging to tolerate drug therapy.^[10–14]^ Although the outcomes of patients with post-infarction HF have marginally improved with the development of pharmacological and non-pharmacological therapies, their all-cause mortality, cardiovascular event rates, and re-hospitalization rates remain high.^[15,16]^

Traditional Chinese medicine (TCM) has shown lower adverse effects in reducing HF symptoms and improving patients’ quality. As a common TCM for the treatment of angina pectoris in coronary heart disease, Compound Xueshuantong Capsule (CXSTC) has been clinically used for >20 years. Its main components include Xuan Shen (*Scrophularia ningpoensis*), Huang Qi (*Astragalus membranaceus*), San Qi (*Panax notoginseng*), and Dan Shen (*Salvia miltiorrhiza*), which are interrelated and form a complex and complementary whole.^[17]^ CXSTC has shown anti-thrombotic and myocardial protective effects in a rat model.^[18,19]^ Clinical studies have shown CXSTC can reduce the number of angina attacks,^[20]^ alleviate clinical symptoms,^[21–23]^ improve N-terminal pro-B-type natriuretic peptide levels and other efficacy indices,^[24]^ improve lipid metabolism,^[25,26]^ inhibit platelet activation, improve endothelial cell function,^[27]^ and reduce levels of serum ultrasensitive C-reactive protein and vascular endothelial growth factor after PCI in patients with coronary artery disease.^[28]^ Based on the above studies, we hypothesized that early intervention of acute MI (AMI) with CXSTC based on modern standardized medical therapy might be more effective in protecting the myocardium.

This trial will be conducted as a multi-center, randomized, double-blind, placebo-controlled clinical study, aiming to provide a clinical evidence-based basis for the prevention and treatment of post-infarction HF using TCM.

## 2. Methods

### 2.1. Study design

**Figure 1.**
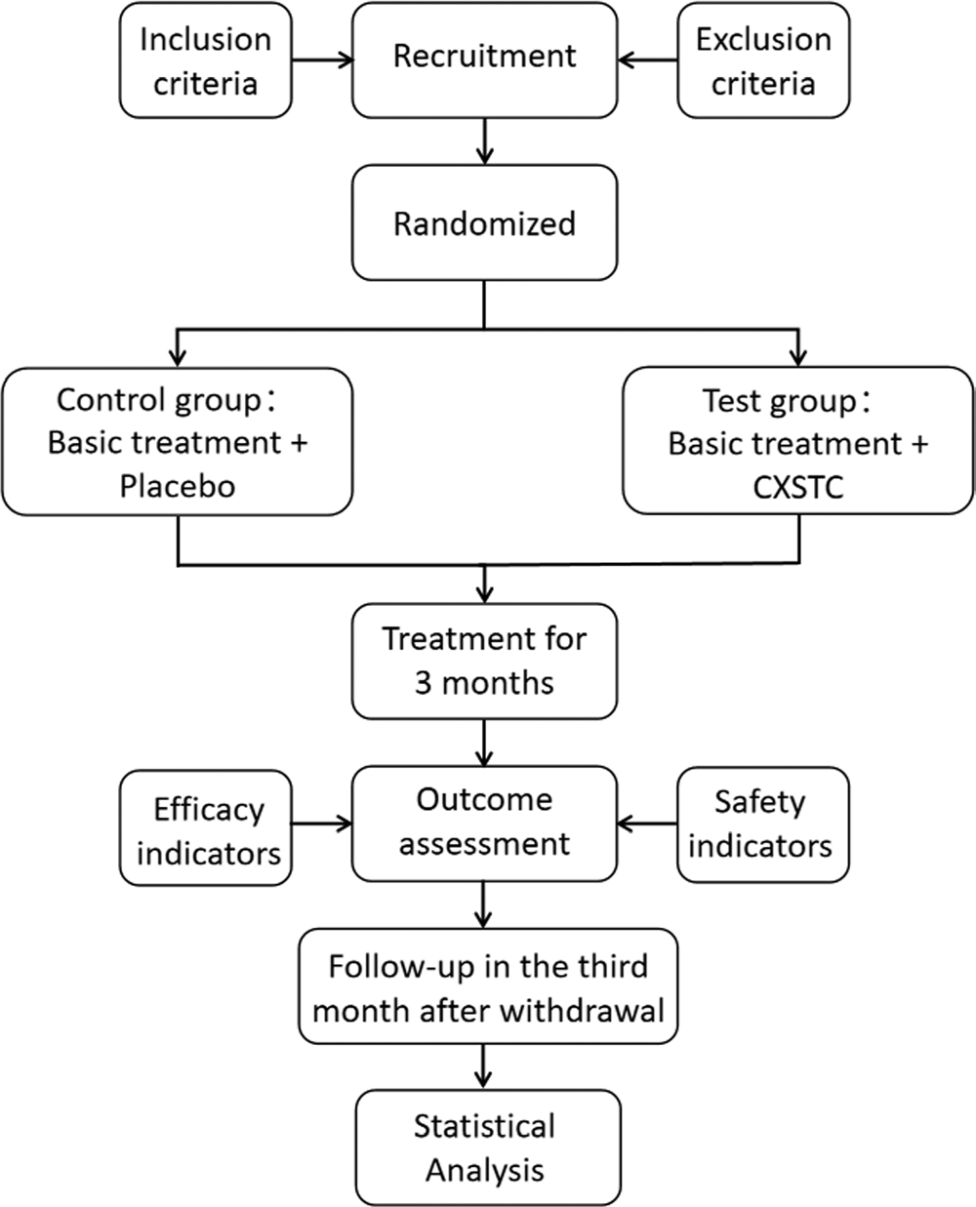
Flowchart of the study. CXSTC = Compound Xueshuantong Capsule.

**Figure 2:**
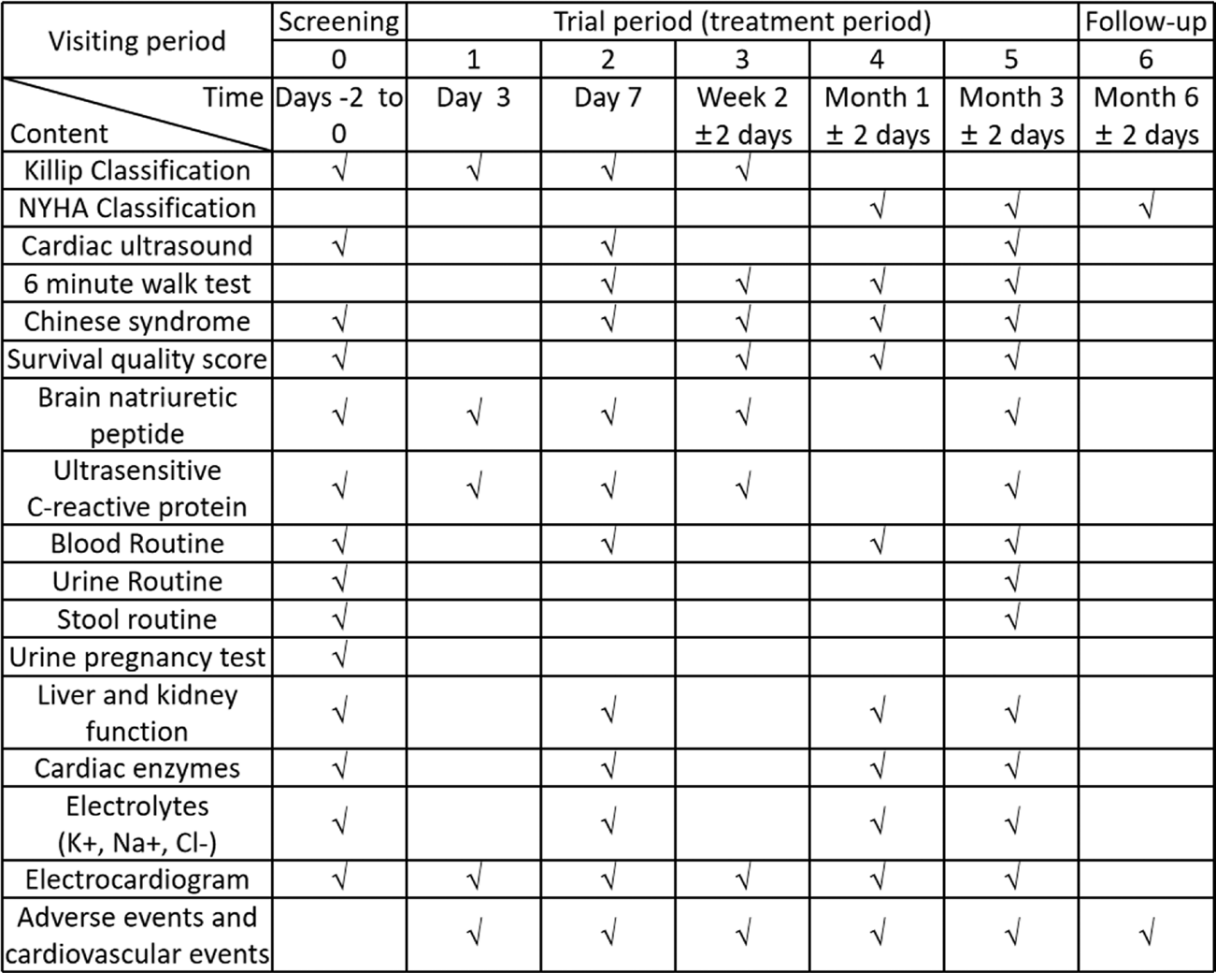
. The schedule of enrollment, interventions, and assessments. NYHA = New York Heart Association.

This will be a multi-center, randomized, double-blind, placebo-parallel controlled trial. Between January 2020 and December 2023 in 9 centers including The First Affiliated Hospital of Guangzhou University of Traditional Chinese Medicine, the Affiliated Hospital of Liaoning University of Traditional Chinese Medicine, Yueyang Integrated Hospital of Traditional Chinese and Western Medicine Affiliated to Shanghai University of Traditional Chinese Medicine, the First Affiliated Hospital of Guangxi University of Traditional Chinese Medicine, Hainan Hospital of Traditional Chinese Medicine, Maoming Hospital of Traditional Chinese Medicine, Dongguan People’s Hospital, the Second Affiliated Hospital of Guangzhou Medical University, and Beihai People’s Hospital, a total of 300 patients diagnosed with AMI and undergoing PCI within 12 hours of diagnosis, with the Chinese medicine diagnosis of “Qi-Yin deficiency and blood stasis,” will be randomly divided into 2 groups: the control group that will be administered conventional Western medicine plus placebo, and the experimental group that will be administered CXSTC along with the conventional Western medicine. Figure 1 shows a flow chart of the study. Figure 2 presents the schedule of enrollment, interventions, and assessments.

### 2.2. Ethics and registration

This study will be conducted in accordance with the Ethical Principles for Medical Research from the Declaration of Helsinki and other relevant regulations. It has been approved by the Ethics Committee of the First Affiliated Hospital of Guangzhou University of Chinese Medicine and annual review (Ethics No. NO.ZYYECK [2019] 080). Before each patient is enrolled in this study, the investigator will provide a complete and comprehensive description of the purpose, procedures, and possible risks of this study to the patients or their representative and obtain written informed consent. Patients will be made aware of their right to withdraw from the study at any time, and the informed consent will be retained as a clinical study document for review. Patient privacy and data confidentiality will be protected during the study.

China Clinical Trials Registry registration number: ChiCTR1900026545.

### 2.3. Patients

#### 2.3.1. Diagnostic criteria for AMI.

(1) Definition and diagnosis of AMI

Refer to the 2015 Guideline on the diagnosis and therapy of ST-segment elevation MI and the 2016 Guideline on the diagnosis and therapy of non-ST segment elevation acute coronary syndrome.

(2) Killip classification

Refer to the 2015 Guideline on the diagnosis and therapy of ST-segment elevation MI.

#### 2.3.2. Diagnostic criteria for HF.

Refer to the Chinese guidelines for the diagnosis and treatment of HF 2018.

#### 2.3.3. Diagnostic criteria and efficacy evaluation of TCM.

Refer to the 2018 Guideline on the diagnosis and therapy of AMI by integrative medicine and the 2002 Guiding principles of clinical research on new drugs of Chinese medicines.

#### 2.3.4. Recruiting.

Patients diagnosed with AMI and undergoing PCI within 12 hours of diagnosis at each study center will be screened by the inclusion and exclusion criteria and then invited by the clinician after providing a complete and comprehensive description of the purpose, procedure, and possible risks of the study to the patient or their representative and obtaining written informed consent.

#### 2.3.5. Inclusion criteria.

(1) Compatible with the diagnosis of AMI.
(2) Patients underwent PCI within 12 hours of infarction or PCI after emergency thrombolysis.
(3) Patients with an ejection fraction of >50%.
(4) Patients who meet the criteria of Chinese medicine identification of Qi and Yin deficiency and blood stasis.
(5) Age between 18 and 75 years, regardless of sex.
(6) Patients who voluntarily accept the corresponding treatment and sign the written informed consent.

#### 2.3.6. Exclusion criteria.

(1) Those with AMI Killip classification grade II or above.
(2) AMI combined with serious complications such as cardiogenic shock (not corrected by conventional treatment) combined with mechanical complications.
(3) Severe arrhythmias (such as rapid-type atrial fibrillation, ventricular tachycardia, and high atrioventricular block).
(4) Those with a previous history of chronic HF or old MI.
(5) Patients with combined cardiomyopathy, wind heart disease, severe heart valve disease, pericardial tamponade, hypertensive emergencies, pulmonary hypertension, severe chronic obstructive pulmonary disease or acute exacerbation of asthma, severe infections, etc.
(6) Patients with severe primary diseases of the liver (alanine aminotransferase level ≥3 times the upper limit of normal value), kidney (creatinine ≥3 mg/dL or 265 μmol/L), endocrine system, and hematopoietic system, or psychiatric patients.
(7) Patients with combined malignancies.
(8) Patients who are allergic to multiple drugs and foods or with a known allergy to the ingredients of CXSTC.
(9) Patients who cannot take care of themselves or cannot take oral medication.
(10) Patients who have participated in other clinical studies within the previous 3 months.
(11) Patients with a suggested or confirmed alcohol or drug abuse history.
(12) Pregnant or lactating women.
(13) Patients with a history of cerebrovascular accident and major surgery and trauma in the previous 6 months.

#### 2.3.7. Additional exclusion criteria.

(1) Patients who do not cooperate with randomization, do not take any trial drug after randomization, or use very little drug (<10%).
(2) Patients who use the prohibited combinations of treatments or drugs, which would affect the effectiveness and safety determination.
(3) No data after randomization.

The case will be judged for exclusion after a discussion between the principal investigator, data manager, statistical analysis expert, and sponsor during a blinded review.

#### 2.3.8. Criteria for discontinuation of study.

Trial discontinuation is defined as stopping the entire trial midway through a clinical trial that has not been completed according to the protocol. The trial shall be discontinued if one of the following occurs.

(1) Serious safety issues arise during the trial.
(2) In the course of the trial, it is found that the clinical trial protocol has a major error, evaluating the drug effect is difficult, or serious deviations in the implementation render it difficult to continue to evaluate the efficacy of the drug.
(3) Administrative authorities withdraw the trial for other causes and need to clarify the reasons.

### 2.4. Sample size

Taking α = 0.05 and β = 0.15, a 2-sided test will be used. The estimated sample incidence of P_1_ = 25% for the test group and P_2_ = 43% for the control group will be brought into the following equation:


$$\begin{array}{l} n_{1}=n_{2}=1641.6\times {\left(\frac{u_{\alpha }+u_{2\beta }}{sin^{-1}\sqrt{P_{1}}-sin^{-1}\sqrt{P_{2}}}\right)}^{2} \end{array}$$

### 2.5. Randomization and blinding

The randomization scheme provided by the Department of Statistics, Guangzhou University of Traditional Chinese Medicine. A stratified randomization method will be used. Statistical analysis system software will be applied to generate a randomized allocation of the treatments received by the patients, that is, the treatment assignments corresponding to the running numbers 001 to 300 will be listed, and the study subjects will be randomly divided into 2 groups: the test group and the control group.

The study will have a double-blind design with placebo as the control, and the number of cases in the test and control groups will be 1:1, which will be a secondary blinding done by the Statistics Department of Guangzhou University of Traditional Chinese Medicine. The trial drug and placebo will be provided by Guangdong Zhongsheng Pharmaceutical Co., Ltd. and packaged according to the random assignment and blinding methods. The blind codes will be sealed in duplicate and separately stored in the drug clinical trial institution and the sponsoring unit in charge of this study.

### 2.6. Interventions

The basic treatment will involve following the guideline-guided drug regimen for post-PCI AMI and HF.

Refer to China Society of Cardiology of Chinese Medical Association 2015 Guideline on the diagnosis and therapy of ST-segment elevation MI, 2016 Guideline on the diagnosis and therapy of non-ST segment elevation acute coronary syndrome, and 2016 ESC Guidelines for the Diagnosis and Treatment of Acute and Chronic HF. Drug therapy will be selected based on the severity of the patient’s condition and indications. The indications, doses, and courses of treatment will be strictly formulated according to the guideline recommendations: Dual-anti platelet-therapy, statins, ACEI/angiotensin receptor blockers, β-blockers, anti-ischemic therapy drugs, etc. Those with acute HF after infarction will be treated according to the situation: diuretics, vasodilators, positive inotropic drugs, etc; those with stable acute HF or chronic HF with diuretics, ACEI/angiotensin receptor blockers, β-blockers, aldosterone receptor antagonists, anti-HF treatment drugs, etc.

Test group: basic treatment + CXSTC, 3 capsules/dose, 3 times a day with warm water.

Control group: Basic treatment + placebo, 3 capsules/time, 3 times a day, with warm water.

Duration of treatment: 3 months.

Follow-up: 6 months.

Combined medications: Except for the medications prescribed for the course of the study, the use of TCM (proprietary Chinese medicine, tonics, injections) will be prohibited for all patients; for drugs or other treatments that must be continued for preexisting comorbidities and their symptoms (other than the symptoms of the disease), the name, usage, dosage, and duration of the drug (or other treatment) shall be recorded in detail; in case of comorbidities or symptoms during the study period, all symptomatic treatments will be administered according to clinical routine, and the name, usage, dosage, and time of the drug (or other therapy) shall be recorded in detail; and combined medications and therapies that need to be used during the study in case of adverse events shall be recorded in detail in the case report form.

### 2.7. Outcomes

#### 2.7.1. Efficacy indicators

(1) The main efficacy indicator will be the incidence of HF. The incidence of HF was recorded on days 3, 7, 14, 1 month, and 3 months after drug administration, and after discontinuation of the drug until 3 months.
(2) Secondary efficacy indicators

① Cardiac function classification: before treatment and after drug administration on days 3, 7, 14, 1 month, and 3 months and after discontinuation of the drug until the 3rd month of the examination record.

② Cardiac ultrasound: the examination records shall be recorded before treatment and on days 7 and 3 months after drug administration.

③ Six-minute walk test score: recorded on days 7, 14, 1 month, and 3 months after drug administration.

④ TCM syndrome score: recorded before treatment and on days 7, 14, 1 month, and 3 months after drug administration. The efficacy = (the integral value before treatment − the integral value after treatment)/ the integral value before treatment × 100%); It is assessed at 4 levels: clinical recovery: clinical symptoms or syndrome score decreased by ≥95% from the baseline; marked efficacy: the syndrome score decreased by ≥70% and <95%; efficacy: the syndrome score was a decrease of at least 30% and <70%; invalid: the syndrome score was a decrease of <30%; and worsen: the syndrome score exceeded the baseline after treatment.

⑤ Quality of survival score (the Minnesota living with heart failure questionnaire): recorded before treatment and on days 14, 1 month, and 3 months after drug administration.

⑥ Brain natriuretic peptide level was recorded before treatment and on days 3, 7, 14, and 3 months after drug administration.

⑦ Ultrasensitive C-reactive protein level: recorded before treatment and on days 3, 7, 14, and 3 months after drug administration.

Total efficacy rate = (clinical recovery + marked efficacy + efficacy) cases/a total number of cases × 100%.

#### 2.7.2. Safety indicators.

Routine blood, urine, stool, liver and kidney function, electrolyte monitoring, cardiac enzymology, and electrocardiogram.

#### 2.7.3. Adverse events.

Although adverse effect of CXSTC has not been observed, possible adverse reactions should be concerned, including gastrointestinal reactions, gastrointestinal bleeding, and skin allergy. Any adverse events occurred should be recorded in “Adverse events form” and the treatment process and results also need to be recorded in detail.

#### 2.7.4. Collection of biological specimens.

The patient’s blood, urine and stool samples will be collected before and after intervention, and clearly marked according to the unified format. Then, the samples will be transferred in frozen pipe and stored at −80°C for preservation in the biological sample bank of the First Affiliated Hospital of Guangzhou University of Chinese Medicine. Consents and other documents like the collection and usage of participant data and biological specimens could be inquired from the registry web or the corresponding author.

### 2.8. Study quality control

A multi-center study coordinating committee shall be established, and the study leaders and sponsors of each participating unit shall be the coordinating committee members. The coordinating committee will be responsible for the implementation of the trial and the resolution of trial-related issues. The sponsor commissions the company to be the contract research organization for the trial. The contract research organization shall identify monitors with medical/pharmacological backgrounds and good clinical practice training to supervise the whole process of the clinical trial. In the event of an adverse event or reaction in a clinical trial, the investigator may take necessary measures to protect the safety of the patient according to the condition, record the event, and decide whether to terminate the trial. If a serious adverse event occurs, the clinical trial shall be withdrawn and appropriate treatment measures taken for the patient immediately. Additionally, the same shall be reported to the State Food and Drug Administration department within 24 hours of drug registration, the department of safety supervision and the local provincial drug regulatory administration, sponsor, and the ethics committee. The sponsor shall immediately inform other trial centers and investigators involved in this trial and ensure that all reporting procedures required by laws and regulations are met.

### 2.9. Statistical analysis plan

All data will be statistically analyzed using SPSS 23.0 software. The measurement data will be expressed as mean ± standard deviation to test the normality of the samples. If the distribution is normal and the variances are homogeneous, the *t* test shall be used. The rank sum test will be used if the distribution is not normal or the variances are not equal. Count data will be expressed as percentages and tested by the χ^2^ test or Fisher method. Rank data will be tested by rank sum test; the Cox regression model will be used to observe the relationship between the risk of occurrence of endpoint events and each influencing factor. A 2-sided *P* value < .05 will be set as the significant level.

## 3. Discussion

In the present study, patients with AMI will be studied, and early intervention with the classical Chinese medicine CXSTC will be combined with modern standardized medical therapy. It is hypothesized that CXSTC will further improve the development of ventricular remodeling and HF after AMI. A multi-center, randomized, double-blind, placebo-controlled clinical study shall be conducted using the incidence of HF as the primary efficacy index and ventricular remodeling, TCM syndrome score, quality of survival score, and the incidence of major adverse cardiovascular events as secondary indices.

Thus far, there is no standard large sample of clinical trials to evaluate the clinical studies of CXSTC for the prophylactic treatment of patients with post-infarction HF. Through this clinical study, we hope to provide a clinical evidence-based basis for Chinese medicine prevention and treatment for post-infarction HF. However, we also need to recognize the limitations of this trial. The small number of cases makes it difficult to make a case for the clinical efficacy of CXSTC for the prevention of post-infarction HF. Second, post-infarction ventricular remodeling is a chronic process, and although the patients included in this study will be closely monitored in the short term, it is still difficult to determine the long-term prognosis of the patients with a follow-up period of up to 6 months. Finally, the efficacy and safety of TCM in combination with western drugs need to be further investigated. We hope that further studies will be conducted in the future to provide a clinical evidence base for the use of TCM in the prophylactic treatment of post-infarction HF.

## Acknowledgment

We are grateful to all the researchers and patients who have been involved in this trial.

## Author contributions

**Conceptualization:** Wei Wu.

**Data curation:** Xiao-Dan Yang, Jia-Xi Shi, Wei-can Liao, ChuanJin Luo, QingMin Chu, LiJin Qing.

**Funding acquisition:** Dong-Liang Liu.

**Methodology:** Xin-lin Chen, Jia-Yan Cui, Zheng Jin, Rui Li.

**Supervision:** LiJin Qing, Rong Li, Hui Wu.

**Writing – original draft:** Xiao-Dan Yang, Jia-Xi Shi, Wei-can Liao.

**Writing – review & editing:** Xiao-Dan Yang, Jia-Xi Shi, Wei-can Liao.
